# High thymidylate synthase gene expression predicts poor outcome after resection of hepatocellular carcinoma

**DOI:** 10.1371/journal.pone.0219469

**Published:** 2019-07-10

**Authors:** David B. Donner, Eric K. Nakakura, Alan P. Venook, Heinz-Josef Lenz, Wu Zhang, Jimee Hwang, Emily K. Bergsland, Meng Hsun Lin, Kan Toriguchi, Ricardo J. Antonia, Robert S. Warren

**Affiliations:** 1 Department of Surgery, The University of California San Francisco, San Francisco, California, United States of America; 2 The Helen Diller Family Comprehensive Cancer Center, The University of California San Francisco, San Francisco, California, United States of America; 3 Department of Medicine, The University of California San Francisco, San Francisco, California, United States of America; 4 Department of Preventive Medicine, University of Southern California/Norris Comprehensive Cancer Center, Los Angeles, California, United States of America; 5 Biostatistics Core Facility, The University of California San Francisco, San Francisco, California, United States of America; University of South Alabama Mitchell Cancer Institute, UNITED STATES

## Abstract

**Introduction:**

Prognosis after resection of hepatocellular carcinoma (HCC) is highly variable. Compared to clinicopathologic factors, the use of molecular markers to predict outcome has not been well studied. We investigated the prognostic importance of thymidylate synthase (TS) gene expression and polymorphisms in patients after resection of HCC.

**Methods:**

Patients who underwent complete resection of HCC for whom tissue was available were identified. TS gene expression level and polymorphisms were determined in HCC specimens. Prognostic factors were evaluated using Kaplan-Meier curves and Cox proportional hazard models.

**Results:**

The study included 67 patients. In univariate analysis, variables that negatively influenced survival included TNM stage, microvascular invasion, and high TS expression. For the high TS expression group, median survival was 54 months and 5-year actuarial survival was 47%. For the low TS expression group, median survival was not reached and the 5-year actuarial survival was 91%. In multivariate analysis, only high TS expression remained an independent predictor of poor survival (HR = 10.77, 95% CI 1.36–84.91; *P* = 0.02). TS gene polymorphisms were not associated with TS expression or overall survival.

**Conclusions:**

High TS expression predicts poor outcome after resection of HCC. Molecular markers might be robust predictors of patient outcome after resection of HCC.

## Introduction

Liver cancer is the second leading cause of cancer-related deaths globally, with an incidence of about 850,000 new cases each year. Hepatocellular carcinoma (HCC) accounts for about ninety percent of all cases of primary liver cancer.[1] In the United States, the incidence of HCC has been increasing over the past two decades;[2] there were an estimated 22,620 new cases and 18,160 deaths from HCC in 2009.[3] Overall, the 1- and 3-year survival of patients diagnosed with HCC is 20% and 5%, respectively.[4]

The highly variable prognosis after resection of HCC has led to efforts to identify clinicopathologic factors that would better predict survival. Prognostic factors commonly reported include tumor size, vascular invasion, multifocality, alpha-fetoprotein (AFP) level, Child-Pugh class, bilirubin, and CLIP score.[5] Unlike that of clinicopathologic factors, the importance of molecular markers in predicting outcome in patients after resection of HCC has not been well studied.

In this study, we analyzed the prognostic relevance of thymidylate synthase (TS) mRNA expression in HCC. TS is the target of 5-fluorouracil (5-FU), a pyrimidine anti-metabolic agent that has been used for the treatment of gastrointestinal cancers for nearly 50 years. Indeed, TS was the first chemotherapeutic agent evaluated for efficacy against HCC.[6] 5-FU inhibits TS, the sole *de novo* source of thymidine in cells, thereby shutting down DNA synthesis and inducing cell death. 5-FU by itself, or in combination with other agents, is the most common and effective agent used for the treatment of head and neck, breast, pancreas, and gastrointestinal tract cancers, and the response to 5-FU is at least partially related to the expression of TS.[7–10]

The mechanisms that regulate TS expression are not fully known. However, polymorphisms within the TS gene appear to be important determinants of the level of TS expression, and some polymorphisms affect the probability of a response to 5-FU-based chemotherapy. TS promoter enhancer region (TSER) 2R/3R repeat polymorphisms reflect double (2R) or triple (3R) repeats of a 28-bp sequence upstream of the TS translational start site.[10] Metastatic colorectal and gastric cancers homozygous for the triple repeat (3R/3R) have higher TS expression than those homozygous for the double repeat (2R/R).[11, 12] Poorer response to 5-FU occurs in patients with the 3R allele in colorectal cancer.[13] In addition, a G to C single nucleotide polymorphism (SNP) in the second repeat of the 3R allele has been identified. 3G-containing genotypes (2R/3G, 3C/3G, 3G/3G) are associated with increased transcriptional and mRNA translational activity *in vitro* and *in vivo*, as well as with high TS mRNA levels, poor response to 5-FU based therapy, and poor clinical outcome.[11, 14–17] A third TS polymorphism is a 6-bp deletion in the 3’-UTR. Patients with colorectal cancer homozygous for the TS 3’-UTR 6-bp deletion (-6bp/-6bp) have lower TS mRNA levels than individuals homozygous for the insertion (+6bp/+6bp).[18]

Previous studies reported high TS expression in HCC specimens compared with matched normal liver.[19, 20] The effects of high TS expression in a cohort of Japanese patients with HCC were inconclusive.[21] More recent studies reported the significance of TS expression and gene polymorphisms to 5-FU based therapies and prognosis of Asian patients.[22–24] However, similar studies of patients in non-Asian countries have yet to appear. For this reason, we evaluated TS expression in HCC from patients at the University of California San Francisco (UCSF) who underwent complete resection, and determined the prognostic significance of high TS expression. The frequency and impact of TS polymorphisms on TS expression and prognosis in HCC were also investigated.

## Material and methods

### Patient characteristics

Sixty-seven patients underwent resection of primary HCC at UCSF from 2/09/1990 to 01/09/2007. All specimens were from patients who underwent complete resection (R0) of their liver tumors. No patient received 5-FU or any other systemic therapy before or after resection. The UCSF Committee on Human Research approved the study (CHR Approval# 6172-26943-05).

### Real-time PCR quantification of mRNA expression

Real-time PCR quantified relative TS gene expression in 43 HCC specimens of snap-frozen tissue prepared by placing tissue in liquid nitrogen immediately after resection. Tissue samples were homogenized in Trizol and total RNA was isolated. Total RNA was quantified by measurement of absorbance at OD260 in an Eppendorf spectrophotometer.

cDNA synthesis was conducted with 1 μg total RNA using a SuperScript III first-strand synthesis kit. 1 μl of cDNA synthesis mix (diluted 1:10) was dispensed in triplicate into optical 384-well plates for Taqman RT-PCR. This was mixed with Taqman Universal PCR Mastermix with no AmpErase UNG and Taqman Gene Expression Assays in an end-volume of 10 μl. TS PCR primer and TaqMan probes were obtained as a pre-formulated mixes from Applied Biosystems and used according to the company's assays-on-demand protocol. β-actin was chosen as an endogenous control to normalize expression data. Reactions were performed on an Applied Biosystems ABI Prism 7900. Samples were incubated at 95°C for 15 minutes, followed by 40 cycles at 95°C for 15 seconds, and 60°C for 1 minute. In non-template control replicates of each gene, no false positive was detected. Assays were performed in triplicate.

Taqman PCR results in an increased fluorescent signal, which correlates to the concentration of PCR amplified. When fluorescent signal intensity is plotted versus PCR cycle number, amplification translates into a logarithmic curve which exceeds the nonspecific background after several PCR rounds. The cycle at which the background is exceeded is defined as the threshold cycle. Relative gene expression of TS was determined based on the threshold cycle of the gene of interest and of the internal reference gene. Results were determined as follows: 2^- (deltaCt sample- deltaCt calibrator)^, where deltaCt values of the calibrator and sample were determined by subtracting the Ct value of the target gene from the value of the *β-actin* gene.

### TS genotyping

DNA was extracted from frozen or paraffin-embedded HCC tissues from 67 patients using the QiaAmp kit. Genotyping was done using a PCR-based restriction fragment length polymorphism technique.[12, 16, 18]

### Statistical methods

The non-parametric Mann-Whitney *U* test and the Kruskall-Wallis test were used to test for differences in TS expression between two or more groups. The method of Hothorn and Lausen was used to estimate the cut-point for TS mRNA expression that best separated low and high gene expression groups based on log-rank statistics.[25] The maximally selected rank statistic for this cut-point was calculated using Maxstat statistical software in R language (The R Foundation for Statistical Computing, Version 2.0.0, Vienna, Austria. ISBN 3-900051-00-3, URL: http://cran.r-project.org/).

Overall survival was calculated from the date of hepatic resection to the date of death, or the last date the patient was known to be alive. The Kaplan-Meier method estimated survival probabilities, and the log-rank test compared survival distributions. Cox proportional hazards regression analysis estimated hazard ratios, adjusting for the effect of other potential confounders, which were defined as clinicopathological variables whose p-value was <0.05 in the univariate analysis (microvascular invasion and TNM stage, see Table 1). Statistical significance was set at a *P* value < 0.05. Analyses were carried out using SAS Statistical Software version 9.1.

**Table 1:** Univariate analyses of clinicopathological variables as predictors of overall survival after resection for hepatocellular carcinoma (n = 67).

Variable	n	Median Survival (months)	5-year OS (%)	p-value (Log-rank)
1.Gender				0.3
Males	50	61	53	
Females	17	161	63	
2.Age				0.49
< 60 years	24	40	46	
≥60 years	43	71	60	
3.TNM Stage (AJCC)				**0.02**
I	28	99	72	
II	20	38	46	
III	19	22	38	
4. Cirrhosis of the liver				0.48
Present	37	99	56	
Absent	30	62	55	
5.Histologic Grade				0.48
Well	29	92	57	
Moderate	31	62	49	
Poor	7	22	25	
6.Tumor Number				0.13
Single	43	92	67	
Multiple	24	31	40	
7.Tumor Size (largest size if multi)				0.3
<5 cms	24	99	54	
≥5 cms	43	62	55	
8. Microvascular invasion				**0.002**
Present	23	30	32	
Absent	44	92	66	
9. Surgical margin				0.93
<1 cm	33	71	51	
≥ 1 cm	34	62	59	
10. AFP level (ng/ml)				0.98
<15	23	61	58	
16 to <2000	25	161	56	
≥ 2000	11	92	58	
11. Hepatitis status				0.26
HBs antigen(+)	25	99	68	
anti-HCV antibody (+)	11	43	40	
Negative	28	62	50	

## Results

### Patient and tumor characteristics

Demographic and clinicopathologic characteristics of the patients included in this study are in Table 1. The mean and median age of patients was 64 and 67 years, respectively (range, 26–84), and 75% were male. According to AJCC TNM staging, 28 (42%) patients were stage I, 20 (30%) stage II, and 19 (28%) stage III. Most (55%) patients had evidence of liver cirrhosis, and most (64%) had single tumors. All patients underwent an R0 resection. The surgical margin was < 1 cm in 33 (49%) and ≥ 1 cm in 34 (51%) patients.

### Long-term follow-up and factors influencing survival after hepatectomy

Follow-up with a median duration of 44 months (range, 0.3–157) was available for all surviving patients. The overall median survival of patients was 71 months. Overall, five- and 10-year actuarial survival rates were 57% and 37%, respectively. Multiple variables were evaluated by univariate analysis to determine their impact on survival in the 67 patients who underwent resection of HCC (Table 1). Variables that did not influence survival included age, gender, presence of cirrhosis, histological grade, tumor number, tumor size, surgical margin (< 1 cm vs. ≥ 1 cm), AFP level, and hepatitis status. Variables that negatively influenced survival included TNM stage, the presence of microvascular invasion, and high TS expression (Table 2).

**Table 2:** Multivariate analysis by cox regression.

Variable	Hazard Ratio	95% Confidence Interval	p-value
1. TS gene expression (Low vs. High)	10.77	1.36–84.91	0.02
2. Microvascular invasion (Presence vs Absence)	2.27	0.87–5.94	0.095
3. TNM stage (I vs. II vs. III)	1.59	0.86–2.95	0.1421

**Fig 1:**
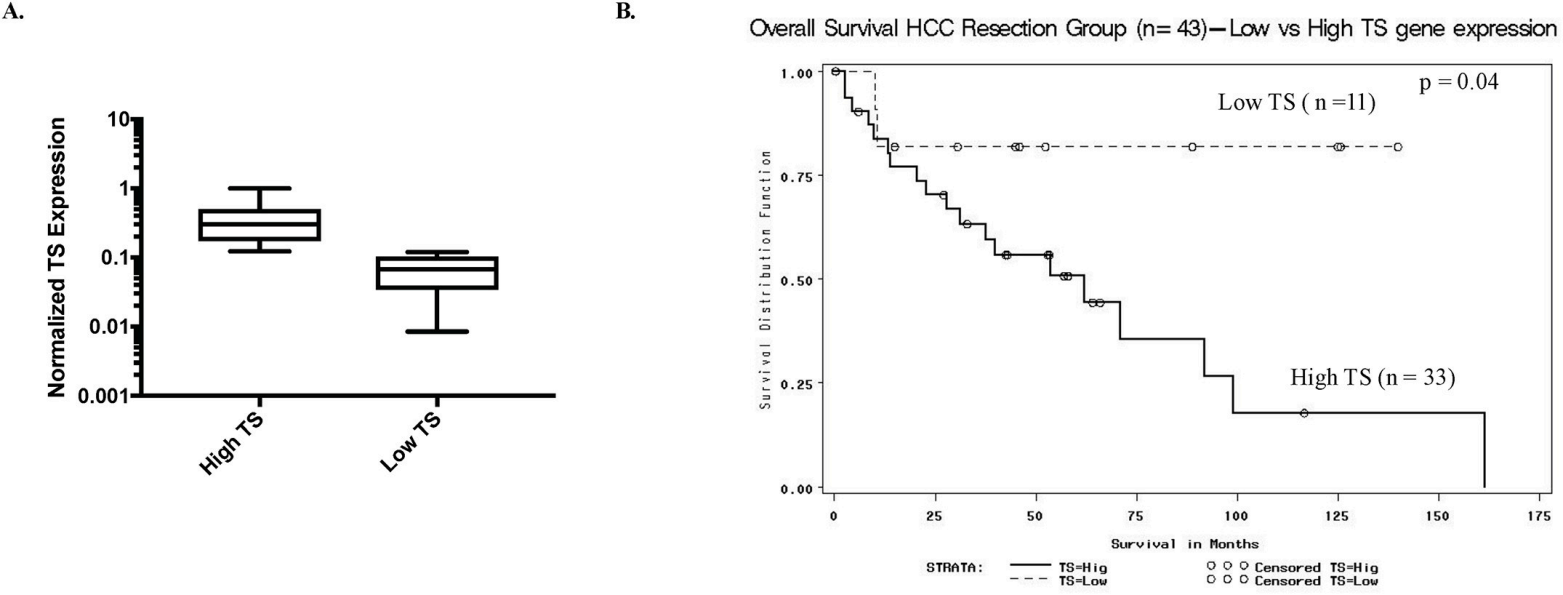
Kaplan Meier plot of patient survival. A. High and low patient TS mRNA levels defined by Maxstat. B. Kaplan Meier plot of patient survival based on mRNA expression characterizes overall survival as a function of TS mRNA levels assayed by qRT-PCR and dichotomized by maximum rank statistics (cutpoint = 0.12), HCC Resection Patients (n = 43).

TS mRNA expression was quantified by RT-PCR of tumor specimens from 44 HCC cases. Data was available for 43 of the 44 cases, because one sample failed to amplify. The range and median of TS mRNA expression was 0.01–1.0 and 0.22, respectively. A cut point of 0.12 was calculated using Maxstat. Fig 1A shows the segregation of the patient mRNAs into high and low thymidylate synthase expression groups; this allowed us to plot patient survival as a function of TS expression in the form a Kaplan-Meier plot (Fig 1B). The median and 5-year overall survival were significantly higher for the group with TS mRNA expression below the cut point (low TS group) than for the group with TS expression above the cut point (high TS group). Although median overall survival was not reached for the low TS group, their 91% 5-year actuarial survival compared favorably, and with very high statistical significance, to a 54-month median and 47% 5-year actuarial survival for the high TS expression group (Fig 1B; *P* = 0.04).

Multivariate analysis, using a Cox proportional hazards model, determined which of the variables that were significant in the univariate analysis were independent predictors of survival (Table 2). Only high TS expression (HR = 10.77, 95% CI 1.36–84.91; *P* = 0.02) remained an independent predictor of worse survival, when adjusted for microvascular invasion and TNM stage.

### Frequency of TS polymorphisms in hepatocellular carcinoma

In some cancers, TS polymorphisms affect TS expression.[10] However, the frequencies of TS polymorphisms in HCC and their association with alterations of TS gene expression have not been studied in patients comparable to that studied here. Therefore, we investigated the frequencies of the TSER 2R/3R, TSER 3R G/C, and TS 3’-UTR 6-bp deletion polymorphisms in 67 HCC samples.

The TSER 2R/3R repeat polymorphism reflects double (2R) or triple (3R) repeats of a 28-bp sequence upstream of the TS translational start site. We observed the following allelic frequencies of TSER 2R/3R repeat polymorphisms: 2R/2R (5%), 2R/3R (43%), and 3R/3R (52%). The TSER 3R G/C polymorphism is a G-to-C single nucleotide polymorphism (SNP) in the second repeat of the 3R allele. We detected the TSER 3R G/C SNP in 48.5% of HCC samples. A third TS gene polymorphism is a 6-bp deletion in the 3’-UTR. We found that 80.6% of HCC samples contained the TS 3’-UTR 6-bp deletion with the following frequencies: +6bp/+6bp (19.4%), +6bp/-6bp (46.3%), and -6bp/-6bp (34.3%).

### TS polymorphisms and gene expression levels

In other cancers, TS polymorphisms influence TS mRNA and protein expression. Evaluation of the effect of TSER 2R/3R repeat polymorphisms on TS gene expression in metastatic colorectal cancer reveals that those homozygous for the triple repeat (3R/3R) have higher TS expression than those homozygous for the double repeat (2R/R). The TSER 3R G/C SNP appears to diminish TS transcription. Patients with colorectal cancer homozygous for the TS 3’-UTR 6-bp deletion (-6bp/-6bp) have lower TS mRNA levels than individuals homozygous for the insertion (+6bp/+6bp). Whether TS polymorphisms affect TS expression in HCC is unknown, leading us to test for such a relationship.

We assayed TS gene expression in HCC samples from 43 patients. Median TS mRNA expression did not differ between patients homozygous for the triple repeat (3R/3R) compared to those with the 2R/2R or the 2R/3R genotype (Table 3; *P* = 0.32). In addition, median TS mRNA levels in HCC patients with the TSER 3R G/C SNP did not differ from those without it (Table 3; *P* = 0.53). Finally, median TS mRNA levels did not differ between patients homozygous for the TS 3’-UTR 6-bp insertion (+6bp/+6bp) and patients heterozygous (+6bp/-6bp) or homozygous (-6bp/-6bp) for the deletion (Table 3; *P* = 0.61). None of the clinicopathologic variables were associated with the level of TS gene expression or the presence of the TS polymorphisms (results not shown).

**Table 3:** Association between TS gene expression levels and polymorphisms in the 5’UTR and 3’UTR.

5'UTR genotype Median (range)		p-value	3'UTR genotype Median (range)		p-value
3R/3R	2R/2R-2R/3R		ins/ins	ins/del, del/del	
0.17 (0.06–1.00)	0.29 (0.01–1.00)	0.34	0.16 (0.04–0.70)	0.23 (0.01–1.00)	0.4
3RG	Non-3RG				
0.23 (0.01–1.00)	0.16 (0.06–1.00)	0.51			

### TS polymorphisms and clinical outcome in hepatocellular carcinoma

**Fig 2:**
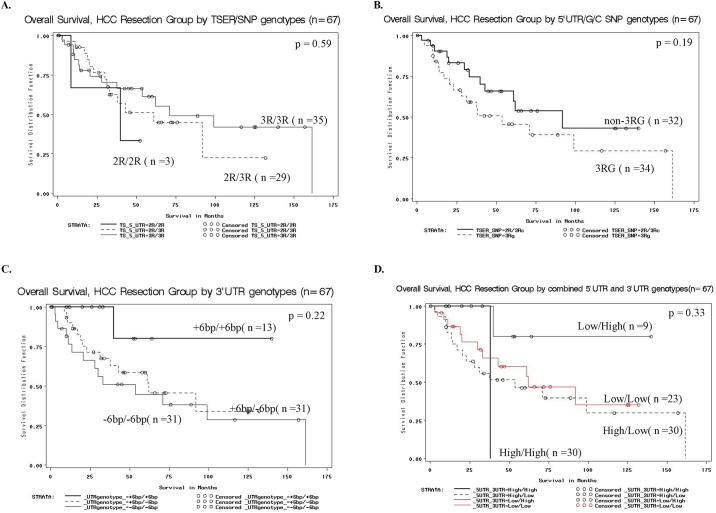
**(A)** Overall Survival by 5'UTR enhancer region polymorphisms, HCC Resection patients (n = 67) (**B**) Overall Survival as a function of TS 5'UTR G/C SNP genotypes HCC Resection Patients (n = 67) **(C)** Overall Survival as a function of TS 3'UTR genotypes HCC Resection Patients (n = 67) **(D)** Overall Survival by TS combined 5'UTR/ 3'UTR polymorphisms, HCC Resection Group (n = 67); Low 5'UTR = 2R/2R + 2R/3C + 3C/3C; Low 3'UTR = +6bp/-6bp + -6bp/-6bp; High 5'UTR = 3G/3G + 3G/3C + 2/3G; High 3'UTR = +6bp/+6bp.

In patients with colorectal cancer, TS polymorphisms predict response to 5-FU-based chemotherapy and influence survival.[10] Whether TS polymorphisms are associated with overall survival in patients after resection of HCC who did *not* receive adjuvant therapy is not known. In this study, repeats of a 28-bp sequence in the 5’UTR, a G/C SNP in the 5'UTR enhancer region, and a 6-bp deletion in the TS 3'UTR were genotyped in 67 HCC cases and their relationship to patient survival was evaluated. Overall survival did not differ among the 2R/2R, 2R/3R, and 3R/3R genotypes (*P* = 0.59)(Fig 2A), nor did overall survival differ in patients with or without the TSER 3R G/C SNP (*P* = 0.26)(Fig 2B). The relationship of 6-bp deletions in the 3'UTR of TS to patient survival was also evaluated. Median survival was not reached for the insertion group and was 61 months for the deletion group. The 5-year survival rates were 67% and 52%, respectively (Fig 2C). Overall survival did not differ among patients with the TS 3’-UTR 6-bp insertion (+6bp/+6bp) compared to those heterozygous (+6bp/-6bp) or homozygous (-6bp/-6bp) for the deletion (*P* = 0.11). Finally, we tested for a relationship of combined genotypes to overall survival (Fig 2D). The median survival for the low 5'UTR/high 3'UTR subgroup was not reached. Median survival was 62 months for the low 5'UTR/low 3'UTR subgroup, 54 months for the high 5'UTR/low 3'UTR subgroup, and 38 months for the high 5'UTR/high 3'UTR group. The 5-year survival rate was 80% for the low 5'UTR/high 3'UTR group, 60% for the low 5'UTR/low 3'UTR subgroup, and 46% for the high 5'UTR/low 3'UTR. There were no 5-year survivors for the high 5'UTR/high 3'UTR subgroup. The differences in 5 year survival for the combined genotypes did not reach statistical significance, but may suggest a trend.

Based on *in vivo* studies, carriers of the TS 5'UTR 2R/2R, 2R/3C, and 3C/3C polymorphisms have been classified as putative low TS genotypes, whereas those with 2R/3G, 3C/3G and 3G/3G have been classified as putative high TS genotypes. We compared the actuarial survival rates of these groups, and found that the median and 5-year overall survival rates were 92 months and 66%, respectively, for the low TS group, compared to 54 months and 46% for the high TS genotype; however, this difference was not statistically significant (p = 0.19).

## Discussion

We have shown that TS expression predicts survival in patients after resection of HCC. Patients bearing HCC tumors expressing high levels of TS had poorer overall survival. Importantly, although HCC specimens express high TS levels compared with matched normal liver,[19, 20] this is the first study to demonstrate an association between high TS expression and poor outcome in patients after resection of HCC. TS is a predictive and prognostic marker in colorectal, gastric, and other tumors.[26–28] Our findings suggest that TS expression may have prognostic significance in patients undergoing complete resection of HCC.

In contrast to our findings, Nii et al. found no significant difference in overall survival in patients with high versus low TS mRNA expression in HCC, although there was a “tendency of better prognosis” in the high TS group.[21] Moreover, Okano et al. found no significant difference in overall survival time between patients with high and low levels of TS.[22] There are important differences between those studies and ours. Nii et al. and Okano et al. segregated patients into high and low expression groups based on the median values of TS mRNA expression, an arbitrary method. We used maximally selected log-rank statistics to identify the optimal cutoffs that actually separated tumors into high and low TS mRNA expression groups. Thus, we used a better statistical method to identify the separation of resection patients into two groups with different overall survival. Future studies may reveal that further sub-stratification based on relative TS mRNA expression levels has even more prognostic significance than a simple high vs low TS mRNA level. Another difference is that we isolated mRNA from snap-frozen samples, whereas Nii et al and Okano et al isolated mRNA from formalin-fixed, paraffin-embedded specimens in which mRNA may be susceptible to degradation by ribonucleases. Finally, the Nii and Okano studies were based on patients who underwent liver resection in Japan, whereas our findings are based on patients in the United States. The patient populations in these studies likely differed with respect to ethnicity and environmental exposures. The frequency of TS polymorphisms varies among ethnic populations,[10] and it is possible that TS expression, and the significance of polymorphisms to outcome, might vary between different patient populations.

Because TS is the target of 5-FU, many studies on the pharmacogenomics of TS sought to identify patients likely to respond to 5-FU and experience better survival.[10] Our findings suggest that for HCC, TS expression is an independent prognostic factor *irrespective* of treatment with 5-FU, as shown for other cancers.[29–34] Studies have shown that TS protein and mRNA levels are higher in cervical, breast, kidney, bladder, lung, and gastrointestinal tumors than in their normal counterparts, and high TS expression is associated with poor prognosis *irrespective* of 5-FU treatment.[29–39] The mechanism by which TS contributes to poor outcome in patients with HCC is unclear. However, TS plays a role in DNA synthesis and repair, derangements of which can promote oncogenesis. In addition, TS overexpression in mice promotes hyperplasia and adenoma formation in the endocrine pancreas.[40, 41] Tumors with elevated TS undergo more active cellular proliferation, which is associated with tumor invasiveness and metastasis.[29, 33, 37] Our results suggest that TS may play a role in HCC tumor progression.

We found no statistically significant association of an individual polymorphism with intra-tumoral TS expression or overall survival after resection of HCC. The possibility of three different polymorphisms complicates efforts to understand the effect of each variant on TS expression and outcome. Indeed, only a few studies in other cancers performed haplotype analysis of the three polymorphisms, as done here. In one such study, gastric cancer patients who underwent complete resection and received adjuvant 5-FU therapy were grouped into high (3G/2C, 3G/3C, 3G/3G, +6bp/+6bp) or low (2R/2R, 2R/3C, 3C/3C, +6bp/-6bp, -6bp/-6bp) expression genotypes.[11] Patients with high expression genotypes, or with at least one high expression allele, had significantly worse overall survival. Our similar analysis showed no difference in overall survival of patients after resection of HCC. However, as with gastric and colon cancers, the absence of a G/C SNP in the second repeat of the 3R allele, the non-3RG genotype, had a better long-term outcome, though this was not statistically significant. This finding, and other studies that reported better survival in gastric and advanced colorectal cancer was associated with better overall response to 5-FU based therapy for non-3RG patients, leads us to speculate that a similar relationship might be found in HCC.

Our results do not establish a relationship between 5’UTR polymorphisms and the expression level of TS mRNA in HCC. Some groups have associated the 3R/3R genotype with higher TS mRNA expression compared to 2R/2R or 2R/3R genotypes[17]; others found no such relationship, but did find that cancer tissues with the 3R/3R genotype had higher expression of TS protein than those with the 2R/3R genotype.[42] These results, together our observations, suggest that TS genotyping, without determination of TS mRNA and protein expression levels, may be insufficient to predict patient responses to 5-FU-based chemotherapy.

We found lower median TS mRNA expression for the non-3RG group than for the 3RG group in HCC, although this was not statistically significant. The polymorphism may consist of two (2R) or three (3R) repeats of the 28-bp sequence.[16, 17] The 3R genotype has been associated with higher TS mRNA and protein expression than the 2R genotype. Two USF family E-box consensus elements are in the tandem repeats of the 3R genotype, whereas only one is in the 2R genotype.[18] These elements bind USF proteins with the additional element in the 3R genotype conferring greater transcriptional activity than the 2R genotype, which may explain the higher TS mRNA expression observed in the 3RG compared to the 2RG group.

In some cancers, –6bp/-6bp and the –6bp/+6bp polymorphisms associate with low TS gene expression, whereas the +6bp/+6bp polymorphisms associate with high TS expression. Contrary to reports that the 6bp insertion genotype predicts shorter overall survival than the 6bp deletion group,[43, 44] in our study, outcomes tended to be better for the 6bp insertion group. This could be attributed to linkage disequilibrium between the 6bp insertion group and the non-3RG group. We found significant predominance of 6bp insertion cases in the non-3RG compared to the 3RG group (75% vs. 25% respectively; p = 0.05), which is similar to observations made in studies of esophageal [27] and rectal cancer.[45] Thus, it is difficult to ascertain if the effect on outcome can be ascribed to the 3'UTR polymorphism or the non-3RG genotype, which tended to have a better outcome than the 3RG group. The association of the 3’UTR polymorphism with the non-3RG group may also explain why median TS mRNA levels in the 6bp insertion group are lower than in the deletion group, in contrast to reports that the 6 bp insertion at nucleotide 1494 in the 3'UTR is associated with high TS gene expression. The 6 bp deletion in the 3'UTR has been associated with decreased mRNA instability and lower tumor TS expression.[18, 45]

There are limitations to this study, which is a retrospective analysis from a single institution, and subject to selection and referral bias. This study involved a discrete number of patients, making it possible that unrecognized or unique genetic characteristics may have limited its capacity to uncover some associations. For example, the frequency of TS polymorphisms varies among ethnic populations, which undermines attempts to study their effect on TS gene expression and prognosis.[10] Nevertheless, our analysis did identify AJCC TNM stage (which combines characteristics of tumor size, tumor number, vascular invasion, direct invasion, perforation of visceral peritoneum, and regional lymph nodes), microvascular invasion, and high TS expression as prognostic factors in HCC—the first two being important pathologic factors described previously.[46, 47] In this cohort, the size of the surgical margin did not predict overall survival as described previously.[48]

## Conclusions

The highly variable prognosis after resection of HCC has led to efforts to identify clinicopathologic factors that permit better prediction of survival.[3, 49] Because the optimal management of patients with HCC remains controversial, identifying patients at risk for a poor outcome will be essential for proper stratification in future clinical trials.[50] Prognostic factors commonly reported include tumor size, vascular invasion, multifocality, alpha-fetoprotein (AFP) level, Child-Pugh class, bilirubin, and CLIP score.[47] However, the importance of molecular markers in predicting outcome in patients after resection of HCC has not been well studied. In this context, our study is valuable because it shows that TS gene expression may be an independent predictor of survival in patients after resection of HCC. This finding suggests the importance of molecular markers, in addition to pathologic staging, in HCC. Future studies involving global gene expression analysis with the relevant clinical data are necessary to identify which genes are the best markers of HCC prognosis.

## References

[1] Llovet JM, Zucman-Rossi J, Pikarsky E, Sangro B, Schwartz M, Sherman M, et al Hepatocellular carcinoma. Nat Rev Dis Primers. 2016;2:16018 10.1038/nrdp.2016.18 . 27158749

[2] Blum HE. Hepatocellular carcinoma: therapy and prevention. World journal of gastroenterology: WJG. 2005;11(47):7391–400. 10.3748/wjg.v11.i47.7391 . 16437707 PMC4725160

[3] Schwartz M, Roayaie S, Konstadoulakis M. Strategies for the management of hepatocellular carcinoma. Nature clinical practice Oncology. 2007;4(7):424–32. 10.1038/ncponc0844 . 17597707

[4] Choti MA. Surgical management of hepatocellular carcinoma: resection and ablation. Journal of vascular and interventional radiology: JVIR. 2002;13(9 Pt 2):S197–203. . 12354837 10.1016/s1051-0443(07)61787-4

[5] Masuzaki R, Omata M. Treatment of hepatocellular carcinoma. Indian journal of gastroenterology: official journal of the Indian Society of Gastroenterology. 2008;27(3):113–22. . 18787282

[6] Pinedo HM, Peters GF. Fluorouracil: biochemistry and pharmacology. Journal of clinical oncology: official journal of the American Society of Clinical Oncology. 1988;6(10):1653–64. 10.1200/JCO.1988.6.10.1653 . 3049954

[7] Danenberg PV. Pharmacogenomics of thymidylate synthase in cancer treatment. Frontiers in bioscience: a journal and virtual library. 2004;9:2484–94. . 15353301 10.2741/1410

[8] Ichikawa W, Uetake H, Shirota Y, Yamada H, Nishi N, Nihei Z, et al Combination of dihydropyrimidine dehydrogenase and thymidylate synthase gene expressions in primary tumors as predictive parameters for the efficacy of fluoropyrimidine-based chemotherapy for metastatic colorectal cancer. Clinical cancer research: an official journal of the American Association for Cancer Research. 2003;9(2):786–91. . 12576451

[9] Longley DB, Harkin DP, Johnston PG. 5-fluorouracil: mechanisms of action and clinical strategies. Nat Rev Cancer. 2003;3(5):330–8. Epub 2003/05/02. 10.1038/nrc1074 . 12724731

[10] Lurje G, Manegold PC, Ning Y, Pohl A, Zhang W, Lenz HJ. Thymidylate synthase gene variations: predictive and prognostic markers. Molecular cancer therapeutics. 2009;8(5):1000–7. 10.1158/1535-7163.MCT-08-0219 . 19383851

[11] Kawakami K, Graziano F, Watanabe G, Ruzzo A, Santini D, Catalano V, et al Prognostic role of thymidylate synthase polymorphisms in gastric cancer patients treated with surgery and adjuvant chemotherapy. Clinical cancer research: an official journal of the American Association for Cancer Research. 2005;11(10):3778–83. 10.1158/1078-0432.CCR-04-2428 . 15897576

[12] Pullarkat ST, Stoehlmacher J, Ghaderi V, Xiong YP, Ingles SA, Sherrod A, et al Thymidylate synthase gene polymorphism determines response and toxicity of 5-FU chemotherapy. The pharmacogenomics journal. 2001;1(1):65–70. . 11913730 10.1038/sj.tpj.6500012

[13] Marsh S, McKay JA, Cassidy J, McLeod HL. Polymorphism in the thymidylate synthase promoter enhancer region in colorectal cancer. Int J Oncol. 2001;19(2):383–6. Epub 2001/07/11. 10.3892/ijo.19.2.383 . 11445856

[14] Gosens MJ, Moerland E, Lemmens VP, Rutten HT, Tan-Go I, van den Brule AJ. Thymidylate synthase genotyping is more predictive for therapy response than immunohistochemistry in patients with colon cancer. International journal of cancer Journal international du cancer. 2008;123(8):1941–9. 10.1002/ijc.23740 . 18661526

[15] Graziano F, Ruzzo A, Loupakis F, Santini D, Catalano V, Canestrari E, et al Liver-only metastatic colorectal cancer patients and thymidylate synthase polymorphisms for predicting response to 5-fluorouracil-based chemotherapy. British journal of cancer. 2008;99(5):716–21. 10.1038/sj.bjc.6604555 . 18728661 PMC2528158

[16] Mandola MV, Stoehlmacher J, Muller-Weeks S, Cesarone G, Yu MC, Lenz HJ, et al A novel single nucleotide polymorphism within the 5' tandem repeat polymorphism of the thymidylate synthase gene abolishes USF-1 binding and alters transcriptional activity. Cancer research. 2003;63(11):2898–904. . 12782596

[17] Morganti M, Ciantelli M, Giglioni B, Putignano AL, Nobili S, Papi L, et al Relationships between promoter polymorphisms in the thymidylate synthase gene and mRNA levels in colorectal cancers. European journal of cancer. 2005;41(14):2176–83. 10.1016/j.ejca.2005.06.016 . 16182121

[18] Mandola MV, Stoehlmacher J, Zhang W, Groshen S, Yu MC, Iqbal S, et al A 6 bp polymorphism in the thymidylate synthase gene causes message instability and is associated with decreased intratumoral TS mRNA levels. Pharmacogenetics. 2004;14(5):319–27. . 15115918 10.1097/00008571-200405000-00007

[19] Sunaga M, Tomonaga T, Yoshikawa M, Ebara M, Shimada H, Saisho H, et al Gene expression of 5-fluorouracil metabolic enzymes in hepatocellular carcinoma and non-tumor tissue. Journal of chemotherapy. 2007;19(6):709–15. 10.1179/joc.2007.19.6.709 . 18230555

[20] Takahashi T, Yoshida H, Mamada Y, Taniai N, Mizuguchi Y, Shimizu T, et al Profiling of fluorouracil-related genes by microdissection technique in hepatocellular carcinoma. Hepato-gastroenterology. 2007;54(78):1612–6. . 18019677

[21] Nii A, Shimada M, Ikegami T, Harino Y, Imura S, Morine Y, et al Significance of dihydropyrimidine dehydrogenase and thymidylate synthase mRNA expressions in hepatocellular carcinoma. Hepatology research: the official journal of the Japan Society of Hepatology. 2009;39(3):274–81. 10.1111/j.1872-034X.2008.00457.x . 19054147

[22] Okano Y, Kuramochi H, Nakajima G, Katagiri S, Yamamoto M. Elevated levels of mRNAs encoding dihydropyrimidine dehydrogenase and thymidylate synthase are associated with improved survival of patients with hepatocellular carcinoma treated with S-1. Oncol Lett. 2017;14(1):930–6. 10.3892/ol.2017.6241 . 28693254 PMC5494709

[23] Wang X, Sun X, Du X, Zhou F, Yang F, Xing J, et al Thymidylate synthase gene polymorphisms as important contributors affecting hepatocellular carcinoma prognosis. Clin Res Hepatol Gastroenterol. 2017;41(3):319–26. 10.1016/j.clinre.2016.10.012 . 28043790

[24] Zhao H, Zhao Y, Guo Y, Huang Y, Lin S, Xue C, et al Clinical significance of the thymidylate synthase, dihydropyrimidine dehydrogenase, and thymidine phosphorylase mRNA expressions in hepatocellular carcinoma patients receiving 5-fluorouracil-based transarterial chemoembolization treatment. Onco Targets Ther. 2013;6:811–8. 10.2147/OTT.S46498 . 23861589 PMC3704606

[25] Hothorn T, Zeileis A. Generalized maximally selected statistics. Biometrics. 2008;64(4):1263–9. 10.1111/j.1541-0420.2008.00995.x . 18325074

[26] Etienne MC, Chazal M, Laurent-Puig P, Magne N, Rosty C, Formento JL, et al Prognostic value of tumoral thymidylate synthase and p53 in metastatic colorectal cancer patients receiving fluorouracil-based chemotherapy: phenotypic and genotypic analyses. Journal of clinical oncology: official journal of the American Society of Clinical Oncology. 2002;20(12):2832–43. 10.1200/JCO.2002.09.091 . 12065560

[27] Kuramochi H, Tanaka K, Oh D, Lehman BJ, Dunst CM, Yang DY, et al Thymidylate synthase polymorphisms and mRNA expression are independent chemotherapy predictive markers in esophageal adenocarcinoma patients. International journal of oncology. 2008;32(1):201–8. . 18097560

[28] Tsourouflis G, Theocharis SE, Sampani A, Giagini A, Kostakis A, Kouraklis G. Prognostic and predictive value of thymidylate synthase expression in colon cancer. Digestive diseases and sciences. 2008;53(5):1289–96. 10.1007/s10620-007-0008-x . 17934851

[29] Edler D, Hallstrom M, Johnston PG, Magnusson I, Ragnhammar P, Blomgren H. Thymidylate synthase expression: an independent prognostic factor for local recurrence, distant metastasis, disease-free and overall survival in rectal cancer. Clinical cancer research: an official journal of the American Association for Cancer Research. 2000;6(4):1378–84. . 10778966

[30] Edler D, Kressner U, Ragnhammar P, Johnston PG, Magnusson I, Glimelius B, et al Immunohistochemically detected thymidylate synthase in colorectal cancer: an independent prognostic factor of survival. Clinical cancer research: an official journal of the American Association for Cancer Research. 2000;6(2):488–92. . 10690528

[31] Fujiwaki R, Hata K, Nakayama K, Fukumoto M, Miyazaki K. Thymidylate synthase expression in epithelial ovarian cancer: relationship with thymidine phosphorylase expression and prognosis. Oncology. 2000;59(2):152–7. 10.1159/000012153 . 10971175

[32] Nakagawa T, Tanaka F, Otake Y, Yanagihara K, Miyahara R, Matsuoka K, et al Prognostic value of thymidylate synthase expression in patients with p-stage I adenocarcinoma of the lung. Lung cancer. 2002;35(2):165–70. . 11804689 10.1016/s0169-5002(01)00407-x

[33] Nomura T, Nakagawa M, Fujita Y, Hanada T, Mimata H, Nomura Y. Clinical significance of thymidylate synthase expression in bladder cancer. International journal of urology: official journal of the Japanese Urological Association. 2002;9(7):368–76. . 12165018 10.1046/j.1442-2042.2002.00479.x

[34] Suzuki M, Tsukagoshi S, Saga Y, Ohwada M, Sato I. Enhanced expression of thymidylate synthase may be of prognostic importance in advanced cervical cancer. Oncology. 1999;57(1):50–4. 10.1159/000012000 . 10394125

[35] Johnston PG, Lenz HJ, Leichman CG, Danenberg KD, Allegra CJ, Danenberg PV, et al Thymidylate synthase gene and protein expression correlate and are associated with response to 5-fluorouracil in human colorectal and gastric tumors. Cancer research. 1995;55(7):1407–12. . 7882343

[36] Leichman CG. Predictive and prognostic markers in gastrointestinal cancers. Current opinion in oncology. 2001;13(4):291–9. . 11429488 10.1097/00001622-200107000-00013

[37] Mizutani Y, Wada H, Yoshida O, Fukushima M, Nonomura M, Nakao M, et al Significance of thymidylate synthase activity in renal cell carcinoma. Clinical cancer research: an official journal of the American Association for Cancer Research. 2003;9(4):1453–60. . 12684419

[38] Pestalozzi BC, Peterson HF, Gelber RD, Goldhirsch A, Gusterson BA, Trihia H, et al Prognostic importance of thymidylate synthase expression in early breast cancer. Journal of clinical oncology: official journal of the American Society of Clinical Oncology. 1997;15(5):1923–31. 10.1200/JCO.1997.15.5.1923 . 9164203

[39] Shintani Y, Ohta M, Hirabayashi H, Tanaka H, Iuchi K, Nakagawa K, et al New prognostic indicator for non-small-cell lung cancer, quantitation of thymidylate synthase by real-time reverse transcription polymerase chain reaction. International journal of cancer Journal international du cancer. 2003;104(6):790–5. 10.1002/ijc.11014 . 12640689

[40] Chen M, Rahman L, Voeller D, Kastanos E, Yang SX, Feigenbaum L, et al Transgenic expression of human thymidylate synthase accelerates the development of hyperplasia and tumors in the endocrine pancreas. Oncogene. 2007;26(33):4817–24. 10.1038/sj.onc.1210273 . 17297449

[41] Rahman L, Voeller D, Rahman M, Lipkowitz S, Allegra C, Barrett JC, et al Thymidylate synthase as an oncogene: a novel role for an essential DNA synthesis enzyme. Cancer cell. 2004;5(4):341–51. . 15093541 10.1016/s1535-6108(04)00080-7

[42] Kawakami K, Salonga D, Park JM, Danenberg KD, Uetake H, Brabender J, et al Different lengths of a polymorphic repeat sequence in the thymidylate synthase gene affect translational efficiency but not its gene expression. Clinical cancer research: an official journal of the American Association for Cancer Research. 2001;7(12):4096–101. . 11751507

[43] Dotor E, Cuatrecases M, Martinez-Iniesta M, Navarro M, Vilardell F, Guino E, et al Tumor thymidylate synthase 1494del6 genotype as a prognostic factor in colorectal cancer patients receiving fluorouracil-based adjuvant treatment. Journal of clinical oncology: official journal of the American Society of Clinical Oncology. 2006;24(10):1603–11. 10.1200/JCO.2005.03.5253 . 16575011

[44] Huang ZH, Hua D, Li LH. The polymorphisms of TS and MTHFR predict survival of gastric cancer patients treated with fluorouracil-based adjuvant chemotherapy in Chinese population. Cancer chemotherapy and pharmacology. 2009;63(5):911–8. 10.1007/s00280-008-0815-6 . 18704422

[45] Stoehlmacher J, Goekkurt E, Mogck U, Aust DE, Kramer M, Baretton GB, et al Thymidylate synthase genotypes and tumour regression in stage II/III rectal cancer patients after neoadjuvant fluorouracil-based chemoradiation. Cancer letters. 2008;272(2):221–5. 10.1016/j.canlet.2008.07.008 . 18722050

[46] Nathan H, Raut CP, Thornton K, Herman JM, Ahuja N, Schulick RD, et al Predictors of survival after resection of retroperitoneal sarcoma: a population-based analysis and critical appraisal of the AJCC staging system. Ann Surg. 2009;250(6):970–6. Epub 2009/08/01. 10.1097/SLA.0b013e3181b25183 . 19644351 PMC3099434

[47] Tandon P, Garcia-Tsao G. Prognostic indicators in hepatocellular carcinoma: a systematic review of 72 studies. Liver international: official journal of the International Association for the Study of the Liver. 2009;29(4):502–10. 10.1111/j.1478-3231.2008.01957.x . 19141028 PMC2711257

[48] Poon RT, Fan ST, Ng IO, Wong J. Significance of resection margin in hepatectomy for hepatocellular carcinoma: A critical reappraisal. Annals of surgery. 2000;231(4):544–51. Epub 2000/04/05. 10.1097/00000658-200004000-00014 . 10749616 PMC1421031

[49] Lencioni R, Chen XP, Dagher L, Venook AP. Treatment of intermediate/advanced hepatocellular carcinoma in the clinic: how can outcomes be improved? The oncologist. 2010;15 Suppl 4:42–52. 10.1634/theoncologist.2010-S4-42 . 21115580

[50] Kelley RK, Venook AP. Novel therapeutics in hepatocellular carcinoma: how can we make progress? Am Soc Clin Oncol Educ Book. 2013 Epub 2013/05/30. 10.1200/EdBook_AM.2013.33.e137 . 23714481

